# Supplementary material for: Pyramidal cell subtype-dependent cortical oscillatory activity regulates motor learning
Source: Commun Biol. 2021 Apr 22;4:495. doi: 10.1038/s42003-021-02010-7 (PMC8062540; doi:10.1038/s42003-021-02010-7)
Supplement: Supplementary file 2 — Supplementary Information [file 42003_2021_2010_MOESM2_ESM.pdf]

Supplementary Information

Pyramidal cell subtype-dependent cortical oscillatory activity regulates motor learning

Takeshi Otsuka and Yasuo Kawaguchi

Supplementary Information (available in this document)

Supplementary Figures 1–8
Supplementary Tables 1–3

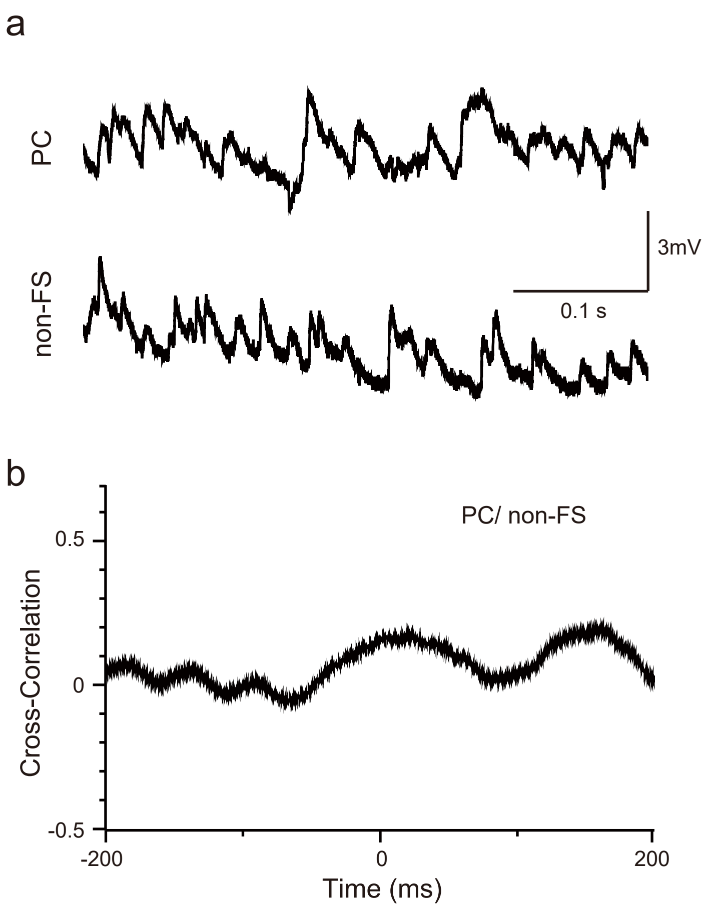

Supplementary Figure 1.

Cross correlation analysis between L5 PCs and non-FS cells during oscillatory activity induced by L2/3 photostimulation. (a) Dual recordings obtained from a L5 PC and non-FS cell. (b) Cross correlogram of membrane potentials of a PC/non-FS cell pair during photostimulation (PC, point of reference). Note that the correlation was weak.

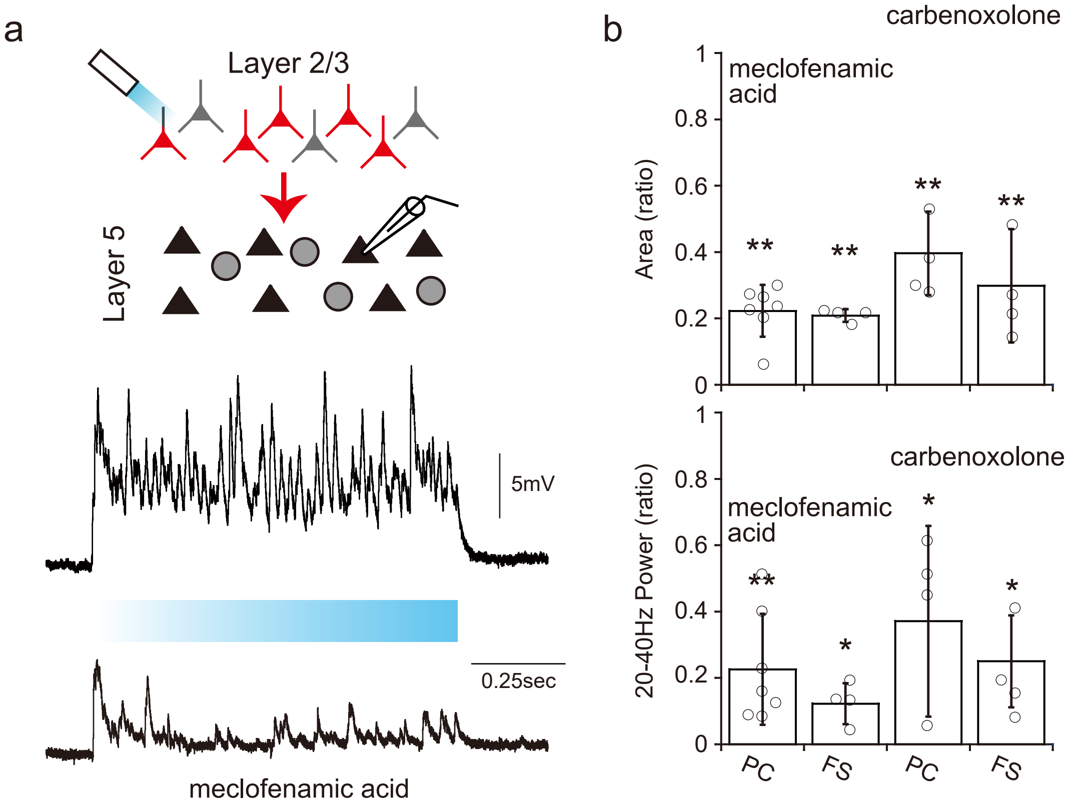

Supplementary Figure 2.

Effects of gap junction blockers on oscillations. (a) Membrane potentials during ramp-shaped light stimulation to L2/3 in the presence and absence of a gap junction blocker (meclofenamic acid, 50 μM) . The gap junction blocker suppressed oscillatory activity in slice preparations. (b) Ratios of integrated area of voltage response during stimulation with blocker to that before application. Two different drugs (meclofenamic acid and carbenoxolone, 50 μM each) were examined to block gap junctions. **, paired t-test, $p<0.01$. Lower: ratios of 20–40 Hz power of membrane potentials during stimulation with blocker to that before application; 7 PCs and 5 FS cells, meclofenamic acid application; 5 PCs and 5 FS cells, carbenoxolone application. Data are expressed as means ± SD. **, $P=0.0017$. *, $p < 0.05$, paired t-test.

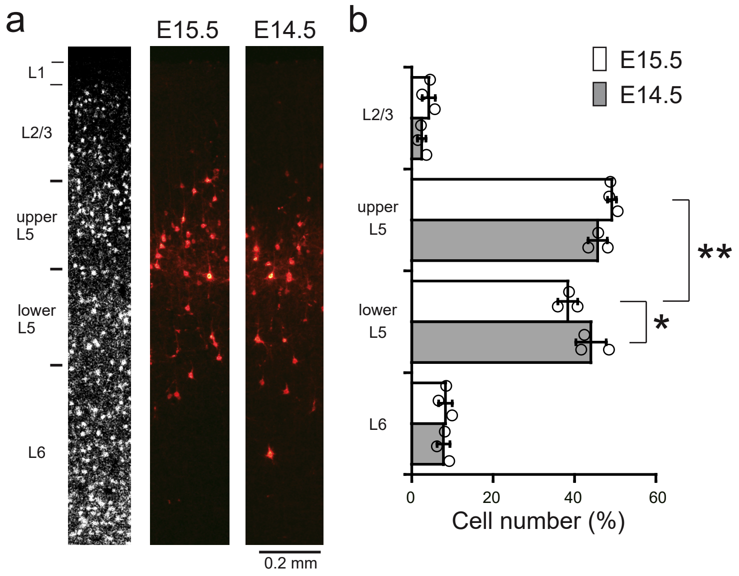

Supplementary Figure 3.

Distribution of cells labeled by *in utero* electroporation at E14.5 and E15.5. (a) coronal section of frontal cortex expressing mCherry by in utero electroporation at E14.5 (right) and E15.5 (middle). Cortical layers were identified by NeuN staining (left). For analysis, L5 was divided into two parts. Brains were fixed at 3 weeks of age. (b) Distribution of labeled cells in individual layers (open bar, E15.5; gray bar, E14.5; n=3 rats for each). *, $p<0.05$, **, $p<0.01$, Welch's t-test. Data are expressed as mean $\pm$ SD.

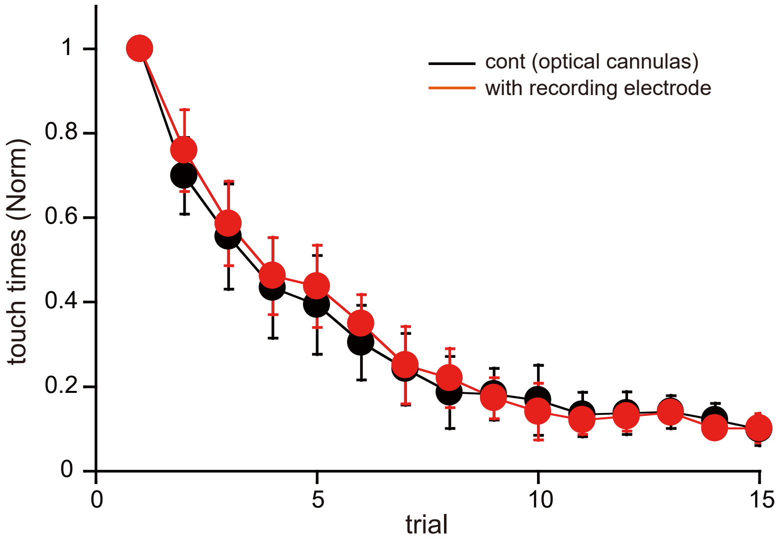

Supplementary Figure 4.

Learning progress monitored by reduction in the number of forelimb touches with the floor during the learning task (5 trials/day). Number of touches was normalized to that of the first trial. Data are expressed as mean ± SD (n=18 and 6 rats implanted optical cannulas (black) and with electrode (red), respectively). No significant difference was found.

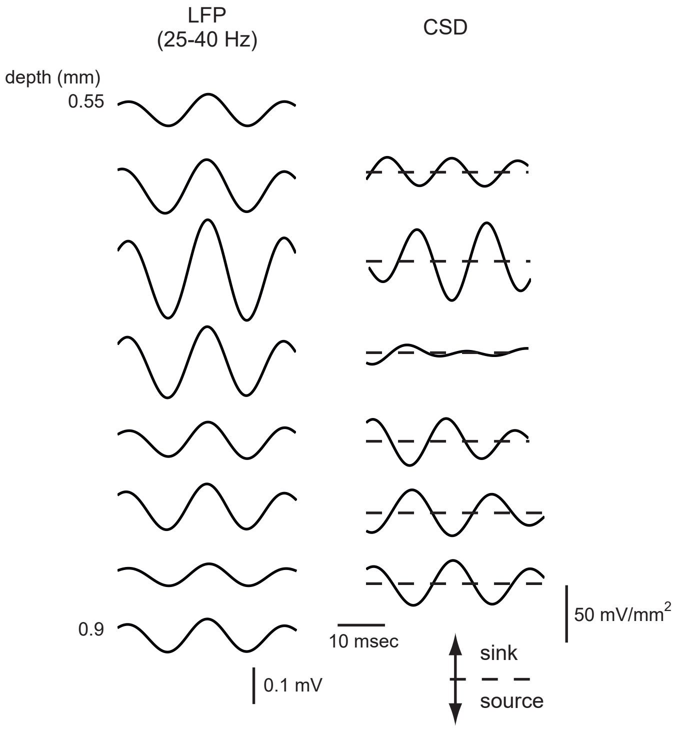

Supplementary Figure 5.

CSD analysis of LFPs. LFPs recorded from the sites positioned at 0.55 to 0.9 mm depth from the pia (left traces) during pattern learning task. LFPs were band-pass filtered at 25-40 Hz. Right, CSD distribution obtained from LFPs shown right traces.

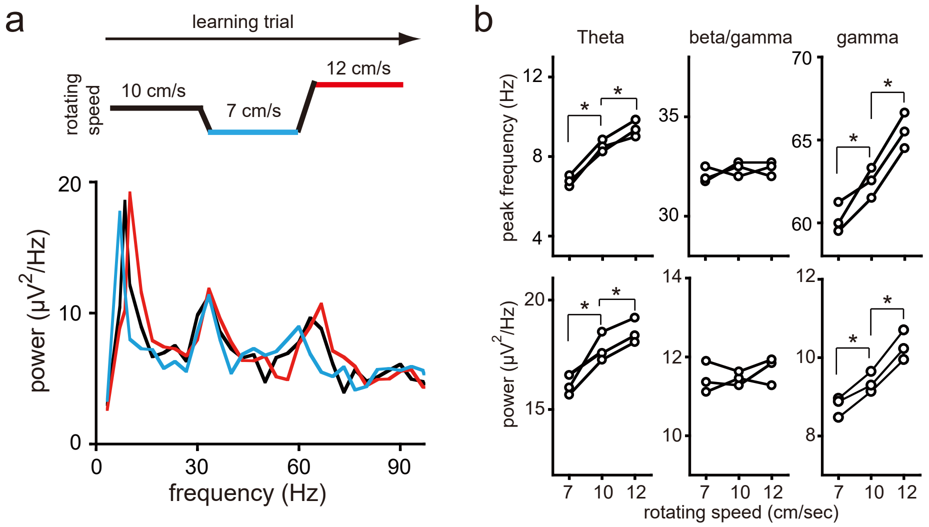

Supplementary Figure 6.

Effects of wheel rotating speed change on LFPs. (a) During a pattern learning task at day1, three different wheel rotating speeds were examined (1 min in duration for each speed, upper inset). Power spectra of LFPs obtained at day1 of learning trials. Colors correspond to rotating speeds shown at upper inset. (b) Peak frequency and power of LFPs at theta, beta/gamma, and gamma frequency bands obtained at different rotating speed (n=3 rats). *, p<0.05, paired t-test.

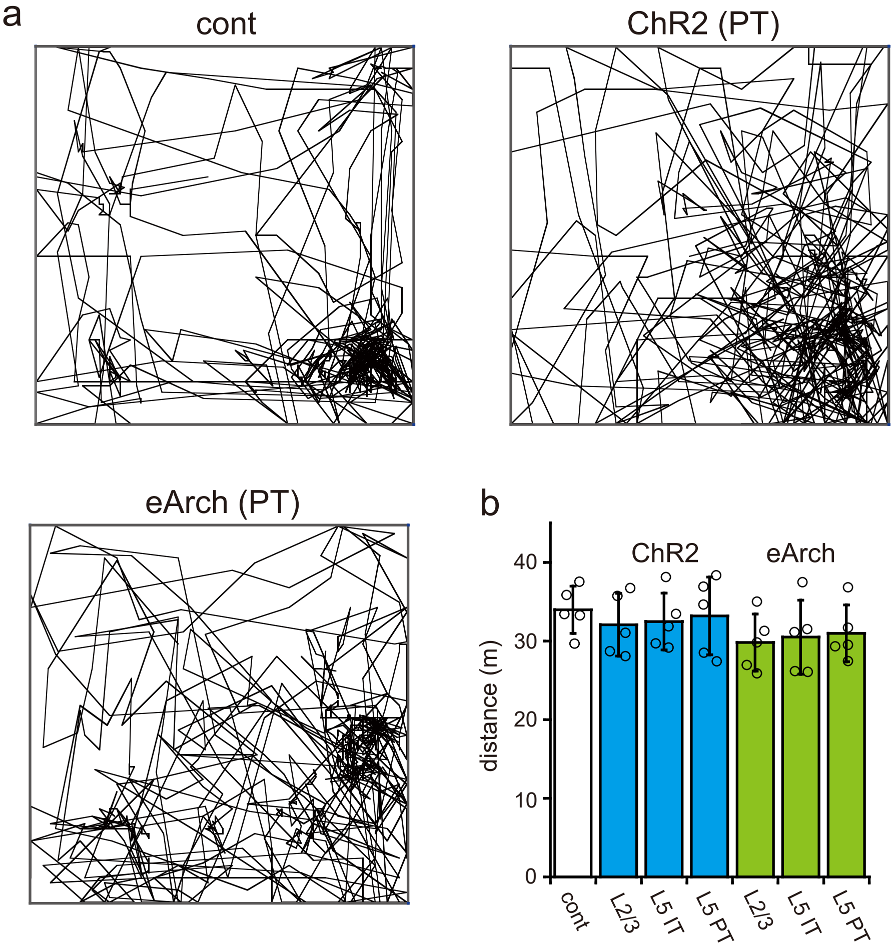

Supplementary Figure 7.

Open field test in a square chamber (50 cm each side). Rats with optical fibers connected to the cannulas were placed in the chamber and their locomotion was monitored for 10 min. The same light stimulation used for the pattern learning task was continuously applied during the test. Positions of the rats were plotted every 1 s. (a) Movement trajectories for control, PT cell-activated (ChR2), and PT cell-inhibited (eArch) rats. (b) Total distances of movement during the test in controls as well as during photo-activation (ChR2; blue) and photo-inhibition (eArch; green) of L2/3 PC, L5 PT, and L5 IT cells (5 rats for each group) were recorded. No significant differences were found (one-way ANOVA, $F_{(6, 28)}$=1.220912, P=0.32525). Data are expressed as means ± SD.

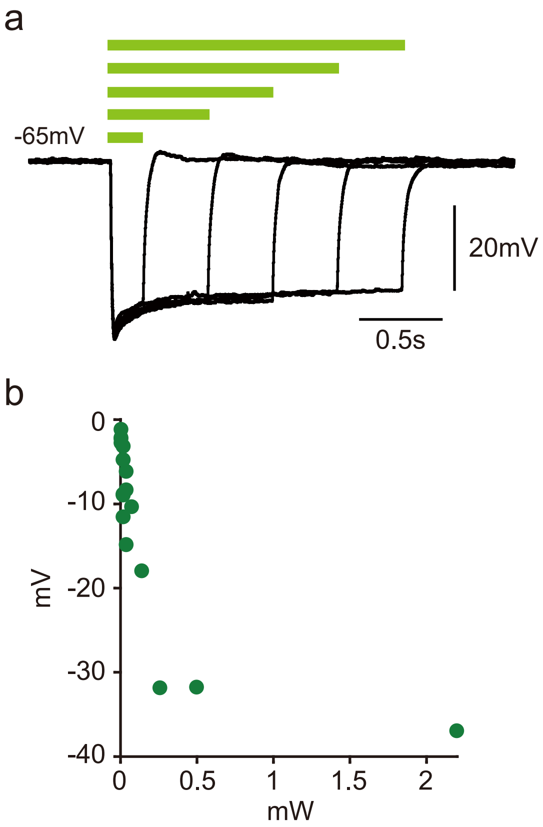

Supplementary Figure 8.

Long-lasting membrane hyperpolarization during eArch activation. (a) Recordings were obtained from L5 PCs expressing eArch in slice preparations. Cells showed sustained membrane hyperpolarization during green light illumination applied through a band-pass filter (510–560 nm wavelength). Green lines indicate durations of photostimulation. (b) Relationship between light intensity and hyperpolarization of membrane potentials. Voltage differences between rest and potentials at the end of photostimulation (duration, 2 s) were plotted against light intensity. Data consist of 6 L5 PCs.

Supplementary Table 1

Basic set of parameter values for the pyramidal cell model

Parameter	Value	Parameter	Value
g_{Na}	70	θ_b	-31
g_K	150	θ_c	-60
$g_{MK\text{-}PT}$	0.54	θ_d	-78
$g_{MK\text{-}IT}$	2	θ_f	-20
g_{Ca}	1.45	θ_h	-45
g_L	0.22	θ_m	-15.5
$g_{pNa\text{-}d1}$	1.1	θ_n	-15.5
$g_{K\text{-}d1}$	10	θ_q	-20
$g_{L\text{-}d1}$	0.2	k_a	-8.5
$g_{A\text{-}d2}$	10	k_b	-11
$g_{Ca\text{-}d2}$	2	k_c	-8.5
$g_{L\text{-}d2}$	0.08	k_d	6.9
V_{Na}	55	K_f	-9.5
V_K	-80	k_h	6.7
V_{Ca}	50	k_m	-16
V_l	-65	k_n	-6
θ_a	-30	k_q	10

Supplementary Table 2

P values in figure 7d (one-way ANOVA with Bonferroni post-hoc tests).

2nd trial; $F_{(3, 46)}$= 22.5365, P<0.0001

Stim	vs. Cont	vs. IT
L2/3	<0.0001	<0.0001
PT	<0.0001	<0.0001

3rd trial; $F_{(3, 46)}$= 23.07729, P<0.0001

Stim	vs. Cont	vs. IT
L2/3	<0.0001	<0.0001
PT	<0.0001	<0.0001

4th trial; $F_{(3, 46)}$= 14.59321, P<0.0001

Stim	vs. Cont	vs. IT
L2/3	<0.0001	<0.0001
PT	0.0006	0.0007

5th trial; $F_{(3, 46)}$= 20.20327, P<0.0001

Stim	vs. Cont	vs. IT
L2/3	<0.0001	<0.0001
PT	<0.0001	<0.0001

6th trial; $F_{(3, 46)}$= 12.01545, P<0.0001

Stim	vs. Cont	vs. IT
L2/3	0.0009	0.0001
PT	0.003	0.0005

7th trial; $F_{(3, 46)}$= 6.78236, P=0.0007

Stim	vs. Cont	vs. IT
L2/3	0.0207	0.0037
PT	0.0739	0.013

8th trial; $F_{(3, 46)}$= 3.22119, P=0.03115

Stim	vs. Cont	vs. IT
L2/3	0.0685	1
PT	0.0811	1

9th trial; $F_{(3, 46)}$= 3.7147, P=0.01803

Stim	vs. Cont	vs. IT
L2/3	0.08	0.053
PT	0.4335	0.2561

10th trial; $F_{(3, 46)}$= 1.77037, P=0.16638

Stim	vs. Cont	vs. IT
L2/3	0.3164	0.951
PT	0.6619	1

11th trial; $F_{(3, 46)}$= 2.8233, P=0.04993

Stim	vs. Cont	vs. IT
L2/3	0.9743	0.0658
PT	1	0.1465

12th trial; $F_{(3, 46)}$= 1.8546, P=0.1509

Stim	vs. Cont	vs. IT
L2/3	0.3329	0.7735
PT	0.6241	1

13th trial; $F_{(3, 46)}$= 1.8063, P=0.15959

Stim	vs. Cont	vs. IT
L2/3	0.2379	1
PT	0.6452	1

14th trial; $F_{(3, 46)}$= 0.50739, P=0.67918

Stim	vs. Cont	vs. IT
L2/3	1	1
PT	1	1

15th trial; $F_{(3, 46)}$= 1.0869, P=0.36433

Stim	vs. Cont	vs. IT
L2/3	1	0.8254
PT	1	1

Supplementary Table 3

P values in figure 8a

One-way ANOVA with Bonferroni post-hoc tests (PT versus Cont, L2/3, or IT)

trial	vs. Cont	vs. L2/3	vs. IT	
2nd	<0.0001	<0.0001	<0.0001	$F_{(3, 45)}$=14.2969, P<0.0001
3rd	<0.0001	0.0009	<0.0001	$F_{(3, 45)}$=11.26715, P<0.0001
4th	<0.0001	<0.0001	<0.0001	$F_{(3, 45)}$=15.1567, P<0.0001
5th	<0.0001	0.0005	<0.0001	$F_{(3, 45)}$=16.68622, P<0.0001
6th	<0.0001	0.0003	<0.0001	$F_{(3, 45)}$=23.21486, P<0.0001
7th	<0.0001	<0.0001	<0.0001	$F_{(3, 45)}$=22.32999, P<0.0001
8th	<0.0001	0.0361	0.0003	$F_{(3, 45)}$=12.11477, P<0.0001
9th	<0.0001	0.1037	0.0001	$F_{(3, 45)}$=10.6898, P<0.0001
10th	0.0025	1	0.0003	$F_{(3, 45)}$=9.178845, P<0.0001
11th	0.0156	1	0.016	$F_{(3, 45)}$=5.302893, P=0.00324
12th	0.0068	1	0.0092	$F_{(3, 45)}$=6.19281, P=0.00324
13th	0.2273	1	0.0328	$F_{(3, 45)}$=3.27903, P=0.02938
14th	0.0261	0.7697	0.0122	$F_{(3, 45)}$=4.45702, P=0.00798
15th	0.0299	1	0.2361	$F_{(3, 45)}$=3.55859, P=0.02146
