# Supplementary material for: Pyramidal cell subtype-dependent cortical oscillatory activity regulates motor learning
Source: Commun Biol. 2021 Apr 22;4:495. doi: 10.1038/s42003-021-02010-7 (PMC8062540; doi:10.1038/s42003-021-02010-7)
Supplement: Supplementary file 3 — Description of Additional Supplementary Files [file 42003_2021_2010_MOESM3_ESM.pdf]

Description of Additional Supplementary Files

File Name: Supplementary Movie 1
Description: Performance of pattern learning task using the forced wheel running system, obtained on day 1 (upper) and day 3 (lower).

File Name: Supplementary Data 1
Description: To Fig. 1f inset: Peak frequencies of oscillation during light stimulation observed in L5 PC and FS cells are given in an Excel file.

File Name: Supplementary Data 2
Description: To Fig. 2c: Time lag of nearest positive peak of correlation between PC/PC and PC/FS cell pair is given in an Excel file.

File Name: Supplementary Data 3
Description: To Fig. 3c and e: Number of PT and IT cells with/without oscillation evoked by light stimulation (upper) and spike number evoked during stimulation in L5 cells (lower) are given in an Excel file.

File Name: Supplementary Data 4
Description: To Fig. 4d: Proportion CTIP2 positive and negative cells among L5 cells expressing mCherry by in utero electroporation at E15.5 and E14.5 is given in an Excel file.

File Name: Supplementary Data 5
Description: To Fig. 6g: Peak frequency band of oscillation between 25~45 Hz during pattern task is given in an Excel file.

File Name: Supplementary Data 6
Description: To Fig. 6h: Absolute peak power of theta, beta/gamma, and gamma (60-70 Hz) frequency band during task through learning days is given in an Excel file.

File Name: Supplementary Data 7
Description: To Fig. 7b and c: Effects of ChR2 stimulation to L2/3 PCs on peak and peak frequency of beta/gamma band power during task are given in an Excel file.

File Name: Supplementary Data 8

Description: To Fig. 6c and Fig. 7d: Normalized number of forelimb touches to the floor during pattern learning tasks with light stimulation in rats expressing ChR2 in L2/3, L5 IT, and L5 PT cells is given in an Excel file.

File Name: Supplementary Data 9
Description: To Fig. 8a: Normalized number of forelimb touches to the floor during pattern learning tasks with light stimulation in rats expressing eArch in L2/3, L5 IT, and L5 PT cells is given in an Excel file.

File Name: Supplementary Data 10
Description: To Fig. 8c: Effects of eArch inhibition on peak of beta/gamma frequency band power during task in rats expressing eArch in L2/3, L5 IT, and L5 PT cells is given in an Excel file.
