# Supplementary material for: Pyramidal cell subtype-dependent cortical oscillatory activity regulates motor learning
Source: Commun Biol. 2021 Apr 22;4:495. doi: 10.1038/s42003-021-02010-7 (PMC8062540; doi:10.1038/s42003-021-02010-7)
Supplement: Supplementary file 15 — Reporting Summary [file 42003_2021_2010_MOESM15_ESM.pdf]

nature research

Corresponding author(s): Takeshi Otsuka

Last updated by author(s): Mar 12, 2021

Reporting Summary

Nature Research wishes to improve the reproducibility of the work that we publish. This form provides structure for consistency and transparency in reporting. For further information on Nature Research policies, see our Editorial Policies and the Editorial Policy Checklist.

Statistics

For all statistical analyses, confirm that the following items are present in the figure legend, table legend, main text, or Methods section.

n/a	Confirmed	
☐	☒	The exact sample size (n) for each experimental group/condition, given as a discrete number and unit of measurement
☐	☒	A statement on whether measurements were taken from distinct samples or whether the same sample was measured repeatedly
☐	☒	The statistical test(s) used AND whether they are one- or two-sided<br>*Only common tests should be described solely by name; describe more complex techniques in the Methods section.*
☐	☒	A description of all covariates tested
☐	☒	A description of any assumptions or corrections, such as tests of normality and adjustment for multiple comparisons
☐	☒	A full description of the statistical parameters including central tendency (e.g. means) or other basic estimates (e.g. regression coefficient) AND variation (e.g. standard deviation) or associated estimates of uncertainty (e.g. confidence intervals)
☐	☒	For null hypothesis testing, the test statistic (e.g. F, t, r) with confidence intervals, effect sizes, degrees of freedom and P value noted<br>*Give P values as exact values whenever suitable.*
☒	☐	For Bayesian analysis, information on the choice of priors and Markov chain Monte Carlo settings
☒	☐	For hierarchical and complex designs, identification of the appropriate level for tests and full reporting of outcomes
☒	☐	Estimates of effect sizes (e.g. Cohen's d, Pearson's r), indicating how they were calculated

Our web collection on statistics for biologists contains articles on many of the points above.

Software and code

Policy information about availability of computer code

Data collection	Data were collected using Axograph (ver. 1.7.6) in slice experiments and Axoscope (ver. 10.4) in in vivo recordings.
Data analysis	Electrophysiological data were analyzed using Axograph (ver. 1.7.6). Statistics were performed on KaleidaGraph (ver. 4.1) and Excell (2019).

For manuscripts utilizing custom algorithms or software that are central to the research but not yet described in published literature, software must be made available to editors and reviewers. We strongly encourage code deposition in a community repository (e.g. GitHub). See the Nature Research guidelines for submitting code & software for further information.

Data

Policy information about availability of data

All manuscripts must include a data availability statement. This statement should provide the following information, where applicable:
- Accession codes, unique identifiers, or web links for publicly available datasets
- A list of figures that have associated raw data
- A description of any restrictions on data availability

Essential source data were uploaded with the manuscript. Additional data of this study are available from the corresponding author upon reasonable request.

Field-specific reporting

Life sciences study design

All studies must disclose on these points even when the disclosure is negative.

Sample size	No sample size calculation was performed. Sample size was determined based on typical sizes in the related previous studies.
Data exclusions	In patch-clamp recordings, neurons that failed to complete a pre-established series of recordings were excluded from the analysis. In in vivo recordings, recordings that were noisy were screened out before proceeding to detailed analysis. For the motor learning task, we excluded rats that showed the decrease of weight continuing for two days. For optogenetic manipulation in in vivo and in vitro experiments, rats expressing ChR2-Venus or eArch-YFP at low level were excluded.
Replication	All electrophysiological, behavioral, and immunohistological experiments were successfully replicated in several animals. Simulation results were confirmed with multiple trials.
Randomization	Not randomized.
Blinding	N.A.

Reporting for specific materials, systems and methods

We require information from authors about some types of materials, experimental systems and methods used in many studies. Here, indicate whether each material, system or method listed is relevant to your study. If you are not sure if a list item applies to your research, read the appropriate section before selecting a response.

Materials & experimental systems

n/a	Involved in the study	
☐	☒	Antibodies
☒	☐	Eukaryotic cell lines
☒	☐	Palaeontology and archaeology
☐	☒	Animals and other organisms
☒	☐	Human research participants
☒	☐	Clinical data
☒	☐	Dual use research of concern

Methods

n/a	Involved in the study	
☒	☐	ChIP-seq
☒	☐	Flow cytometry
☒	☐	MRI-based neuroimaging

Antibodies

Antibodies used	Primary antibodies: rat monoclonal antibody against Ctip2 (ab18465, Abcam), mouse monoclonal antibody against NeuN (MAB377, EMD Millipore)<br>Secondary antibodies: biotinylated anti-rat IgG (BA-4000, Vector), Alexa Flour 350-conjugated streptavidin (S11249, Invitrogen), and Alexa Fluour 488-conjugated streptavidin (S11223, Invitrogen)
Validation	All primary antibodies used in this study were validated by the suppliers. The information is available on their website.

Animals and other organisms

Policy information about studies involving animals; ARRIVE guidelines recommended for reporting animal research

Laboratory animals	Male and female Wistar rats were used in all experiments.
Wild animals	N.A.
Field-collected samples	N.A.
Ethics oversight	All procedures for experiments including behavioual tests were approved by the guideline of the Institutional Animal Care and Use Committee of the National Institutes for Natural Sciences, Japan.

Note that full information on the approval of the study protocol must also be provided in the manuscript.
